# The prospective efficacy of stumble recovery responses in a powered knee prosthesis

**DOI:** 10.3389/frobt.2026.1791652

**Published:** 2026-06-01

**Authors:** Shane T. King, Maura E. Eveld, Karl E. Zelik, Michael Goldfarb

**Affiliations:** 1 Center for Rehabilitation Engineering and Assistive Technology, Mechanical Engineering, Vanderbilt University, Nashville, TN, United States; 2 Biomedical Engineering, Vanderbilt University, Nashville, TN, United States; 3 Physical Medicine and Rehabilitation, Vanderbilt University, Nashville, TN, United States; 4 Electrical Engineering, Vanderbilt University, Nashville, TN, United States

**Keywords:** balance, gait biomechanics, passive prosthesis, perturbation, transfemoral prosthesis control, trip

## Abstract

**Introduction:**

Transfemoral prosthesis users experience heightened fall risk following a trip or stumble when using their conventional, passive prostheses. In a laboratory setting, 54% of all stumble perturbations resulted in a fall for transfemoral prosthesis users using passive prostheses, while able-bodied controls never fell across 190 similar perturbations at a faster walking speed. Shortcomings in the mechanics and control of passive prostheses may contribute to this disparity, including inadequate stance support, poor swing assistance, and the lack of access to certain able-bodied stumble recovery strategies that require positive power at the knee.

**Methods:**

In this work, a powered knee prosthesis was used to provide bimodal stumble recovery behaviors, modeled after able-bodied stumble reflexes, while also providing robust stance support and swing assistance. Three transfemoral prosthesis users underwent a series of treadmill-based obstacle perturbations on both the powered prosthesis and their prescribed, passive prosthesis to compare the stumble recovery outcomes between the two classes of prosthesis.

**Results:**

Results from this preliminary study indicate that the powered prosthesis eliminated knee buckling, improved trunk control, improved base of support adjustment, and reduced hip circumduction. While improvements were consistent across stumble conditions and metrics for two of the three participants, improvements were less consistent with the third participant.

**Discussion:**

Overall, the results suggest that powered stumble recovery-specific behaviors in prosthetic knee prostheses may have promise to improve stumble recovery outcomes for transfemoral prosthesis users.

## 1 Introduction

While tripping or stumbling may present only a minor inconvenience for able-bodied individuals, it carries a more serious risk for transfemoral prosthesis users. Able-bodied individuals rarely fall due to stumbling with previous survey studies reporting only one fall approximately every 3 years across all age groups (Talbot et al., 2005; Verma et al., 2016). However, transfemoral prosthesis users are comparatively at much greater risk of falling and therefore subsequent injury (Hafner et al., 2007; Kahle et al., 2008). Experimental stumble perturbation studies using a treadmill-based perturbation apparatus have found that transfemoral prosthesis users fell during 54% of 24 trials (King et al., 2024), while able-bodied individuals never fell across 190 trials with a similar experimental procedure (King et al., 2019).

The increased fall risk for transfemoral prosthesis users results, in part, from the behavior of commercially-available knee prostheses. While the behaviors of commercial knee prostheses are relatively heterogeneous, nearly all are low impedance and energetically passive (Culver et al., 2022). Low knee impedance enables ballistic swing phase, which mimics the biological mechanism of swing phase in able-bodied individuals (Culver et al., 2022). The energetically passive nature of commercial prostheses avoids the extra bulk, mass, and potential additional control complexity associated with robotic devices (Lee et al., 2019; Lenzi et al., 2018). However, the inability to actively compensate for a swing-phase perturbation limits the ability of the prosthesis to adjust its trajectory in response to disturbances (Crenshaw et al., 2013b; Shirota et al., 2015).

Shortcomings in the fundamental behaviors of commercial, passive prostheses lead to specific functional deficits when recovering from stumble perturbations to the prosthetic side that can result in an increased risk of a fall. In previous work, the authors identified four primary areas of concern (King et al., 2024). First, insufficient stance support resulted in prosthetic knee yielding upon loading following the perturbation, which was the most common failure mode observed in the previous study. Second, inadequate swing extension assistance following the perturbation resulted in increased knee flexion and flexion moment during loading, further exacerbating the inadequate stance support issue and increasing the risk of prosthetic knee yielding. Previous studies have found greater than 30° of knee flexion upon ground contact increased the likelihood of prosthetic knee buckling (Crenshaw et al., 2013b). Third, difficulty initiating swing flexion following the perturbation increased the risk of a fall by causing the foot to remain caught on the obstacle and required participants to compensate by using hip circumduction to move around the obstacle rather than elevating their foot over it by using increased knee flexion, as seen in able-bodied individuals. Hip circumduction can increase the difficulty of the recovery as it increases the time required to recapture center of mass (CoM) support, which may be limited depending on the timing of the perturbation. Fourth, inadequate swing flexion immediately following a perturbation (particularly in early swing phase) limits the individual’s ability to respond by constraining them to a particular recovery strategy known as the lowering strategy, where the swing is terminated early and the limb immediately enters stance phase before the obstacle is crossed in the subsequent stride. In early swing phase, able-bodied individuals utilize the elevating strategy, in which they flex their knee further and cross over the obstacle in the same stride, whereas the lowering strategy is typically used in late swing phase (Eng et al., 1994; Schillings et al., 2000; King et al., 2019; Eveld et al., 2021). Use of the lowering strategy in early swing phase requires the total reversal of swing leg momentum, which can be challenging to complete in time before a fall occurs. However, commercial, passive prostheses’ knee impedance is often too high to allow for enough deflection to successfully clear an obstacle in the manner of the elevating strategy (King et al., 2024).

Some microprocessor-controlled passive prostheses (such as the Ottobock C-Leg) can intentionally increase knee impedance in response to a stumble perturbation to reduce deflection, as they are able to programmatically modify their knee damping from task to task (Blumentritt et al., 2009; Fuenzalida Squella et al., 2018; Hafner et al., 2007; Highsmith et al., 2010; Kahle et al., 2008). By increasing impedance, the prosthesis can attempt to avoid increased knee flexion prior to ground contact and subsequently avoid an increased knee flexion moment leading to knee yielding when entering stance phase. However, this methodology effectively eliminates the ability to perform the elevating strategy used by able-bodied individuals to clear the obstacle in a single stride without requiring the reversal of the swing limb’s forward momentum.

A range of studies have investigated potential rehabilitative and mechatronic interventions to improve stumble recovery and balance perturbation outcomes, either specifically for transfemoral prosthesis users by overcoming the functional deficits of commercial, passive prostheses, or more broadly for those who face mobility impairments. Training approaches such as strength training (Pijnappels et al., 2008) and task-specific gait training such as compensatory step training (Crenshaw et al., 2013a; Grabiner et al., 2012; Rosenblatt et al., 2013) aim to improve an individual’s ability to rapidly manipulate their intact joints to recover from a stumble or loss of balance. Meanwhile, powered knee prostheses have been developed that are capable of mitigating some of the current deficits of commercial, passive prostheses by providing increased stance support and swing assistance (Sup et al., 2009; Rouse et al., 2013; Lawson et al., 2014; Elery et al., 2018; Lenzi et al., 2018; Lee et al., 2019; Tran et al., 2019; Bartlett et al., 2022; Culver et al., 2022). Additional device-based interventions such as perturbation detection (Lawson et al., 2010; Zhang et al., 2011) can provide information to powered prosthesis control systems in order to adjust their behavior in an effort to reject perturbations. While perturbation rejection mechanisms have been implemented in passive, microprocessor-controlled prostheses (Hafner et al., 2007; Kahle et al., 2008; Blumentritt et al., 2009; Highsmith et al., 2010; Fuenzalida Squella et al., 2018) by increasing the prosthetic knee damping upon detection of a perturbation to limit the magnitude of the displacement, recent works have implemented perturbation rejection behaviors in powered knee prostheses (Thatte and Geyer, 2015) and wearable hip and ankle exoskeleton systems (Afschrift et al., 2024; Rajasekaran et al., 2015) to further assist in maintaining balance by enhancing the stabilizing torques in the lower limb joints. In addition to perturbation rejection, studies have implemented obstacle avoidance behaviors in powered prosthesis controllers to avoid the perturbation scenario entirely (Thatte et al., 2019; Gordon et al., 2019; Mendez et al., 2020; Chen et al., 2024; Cheng et al., 2023; Rezazadeh et al., 2019; Zhu et al., 2025). While all of these categories of interventions play a critical role in progressing towards holistic solutions to eliminate balance and stumble recovery deficits for transfemoral prosthesis users, there is a significant lack of implementation and evaluation of powered prosthetic behaviors designed specifically to enhance or assist the reflexive stumble recovery response itself. In this work, the authors explore the prospective efficacy of employing an able-bodied inspired bimodal stumble recovery reflex in response to stumble perturbations.

Specifically, positive power versions of the elevating and lowering strategy were implemented on a powered robotic knee prosthesis. The prospective benefit of the bimodal stumble recovery controller (i.e., utilizing the elevating and lowering strategies) on a powered knee prosthesis was evaluated with three transfemoral prosthesis users with respect to their stumble recovery outcomes using their prescribed, passive prostheses. As such, this paper explores the ability of the powered prosthesis to mitigate the four functional deficits of commercial, passive prostheses previously described.

## 2 Methods

### 2.1 Prosthesis and control

This study employed the Vanderbilt Powered Knee (Lawson et al., 2014). The powered prosthesis utilized a three-stage belt-chain drive system using a brushless, direct current motor (Maxon EC-4 pole 30 mm 36 V) with a transmission ratio of 176:1 and an approximately 85 Nm maximum torque. Onboard sensing included an incremental encoder on the motor, an absolute encoder on the knee joint, an inertial measurement unit (IMU) on the shank, and a full-bodyweight load cell at the base of the prosthesis. The system was battery powered with low-level control running onboard the embedded system and high-level control running on a separate laptop using Simulink Real-Time (Mathworks, Natick, USA) with information communicated via a controller area network interface. More information about the prosthesis can be found in (Lawson et al., 2014).

**FIGURE 1:**
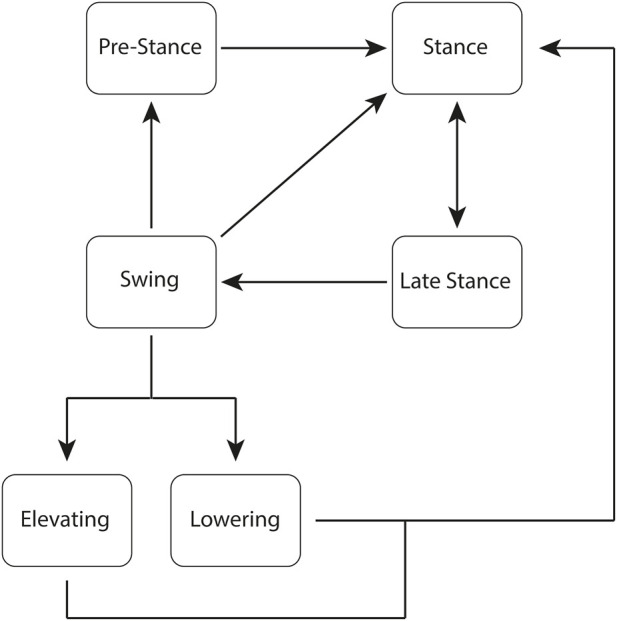
Walking and stumble recovery finite state machine.

A finite state machine-based walking controller (Pre-Stance, Stance, Late Stance, and Swing) was utilized with two additional stumble recovery response states (Elevating and Lowering) branching from the Swing state as shown in Figure 1. The stance states (Pre-Stance, Stance, and Late Stance) and the Lowering state utilize impedance control. Pre-Stance used low gains ( $K_{p}$ : 1.2 Nm/° , $K_{d}$ : 0.2 Nm/(°/s) ) to avoid oscillation while preparing for ground contact. Stance used high gains ( $K_{p}$ : 4.5–5.0 Nm/° , $K_{d}$ : 0.25 Nm/(°/s) ) to provide robust stance support. Late Stance used low gains ( $K_{p}$ : 0.0, Nm/° $K_{d}$ : 0.1–0.3 Nm/(°/s) only in extension) with only intrinsic friction resisting flexion to allow for knee yielding prior to swing. Similar to Stance, Lowering used high gains ( $K_{p}$ : 4.5–5.0 Nm/° , $K_{d}$ : 0.25 Nm/(°/s) ) as it prepared for rapid ground contact following the perturbation before transitioning to swing assistance in the following stride. The Swing ( $K_{p}$ : 4.0–4.5 Nm/° for flexion and 3.5 Nm/° for extension, $K_{d}$ : 0.3–0.35 Nm/(°/s) for flexion and 0.25 Nm/(°/s) for extension) and Elevating ( $K_{p}$ : 6.5–7.0 Nm/° for flexion and 5.0 Nm/° for extension, $K_{d}$ : 0.3 - 0.45Nm/(°/s) for flexion and 0.3 - 0.35Nm/(°/s) for extension) states utilized PD trajectory tracking control to follow real-time generated spline-based swing phase trajectories derived from the initial conditions of swing phase and able-bodied knee kinematic data. The Lowering state ( $K_{p}$ : 6.5 - 7.0Nm/° for flexion and 5.0Nm/° for extension, $K_{d}$ : 0.55Nm/(°/s) for flexion and 0.4Nm/(°/s) for extension) also modified the swing phase spline of the subsequent step to increase knee flexion to allow the user to cross over the obstacle. In the event of a failed elevating strategy (i.e., delayed lowering, where the individual begins to elevate but ultimately abandons it in favor of lowering (Schillings et al., 2000; King et al., 2019)), the same modified peak swing flexion was employed in the subsequent stride. All reported controller gains were tuned empirically to achieve desired state performance, accurate trajectory tracking, and participant comfort.

Stumble perturbation detection was achieved via the IMU using two sets of thresholds, one during knee flexion and one during knee extension due to the different geometric orientation of the foot relative to the obstacle during these phases resulting in a different impulse direction. Flexion phase perturbation detection used a threshold on the axial (with respect to the prosthesis) linear acceleration (<−0.9g) with a low guard threshold on sagittal plane shank angular acceleration $\left(>5{}^{\circ}/s^{2}\right)$ to avoid false positives and a time guard condition to restrict it to swing flexion (<0.18s) . Extension phase perturbation detection used a high threshold on sagittal plane shank angular acceleration $\left(>50{}^{\circ}/s^{2}\right)$ .

In order to select a stumble recovery strategy, a decision algorithm was used based on the post-impact dynamics of the prosthetic limb following the perturbation. The algorithm observed the shank-thigh angular configuration space trajectory, which forms an elliptical path during unperturbed swing phase. Following a perturbation, the decision algorithm observed the deviation of the configuration space trajectory, either internal or external to the elliptical path. Based on observations from prior able-bodied data, outward deviations indicate high forward lower limb momentum and typically result in elevating strategies. Alternatively, inward deviations indicate decreasing or reversed lower limb momentum and typically result in lowering strategies. Specifically, after the perturbation detection, the decision algorithm observed the configuration space trajectory for 20 m before measuring the angle of departure as the Euclidean norm from the original elliptical trajectory. The 20 ms window was selected through pilot testing to ensure consistent estimation of the post-impact dynamics while being as fast as possible to avoid interference with the onset of the reflexive stumble recovery response (i.e., less than 60 m (Eng et al., 1994)). Additionally, the strategy selection decision was biased slightly in the favor of the lowering strategy in an attempt to reduce the likelihood of a delayed lowering responses (i.e., initially elevating before abandoning in favor of lowering). To achieve the bias towards the lowering strategy, an additional angular offset was required for the elevating strategy to be selected. As such, an external deviation of ≥10° with respect to the original unperturbed elliptical path was required in order to select an elevating strategy. Therefore, any deviation that was <10° external or internal to the unperturbed ellipse resulted in a lowering strategy. In very early swing (<0.05 s) , the offset was reduced to 0° since it is extremely unlikely for the lowering strategy to be selected that early in swing phase. More information regarding the decision algorithm and the stumble recovery controller can be found in (King et al., 2026).

### 2.2 Experimental protocol

Three participants from a previous study analyzing the stumble recovery response of transfemoral prosthesis users with their prescribed, passive prostheses were enrolled in this study with a similar protocol. Inclusion criteria for the study included an activity level of K3 or greater and daily use of a passive knee prosthesis. Exclusion criteria were any additional musculoskeletal disorders in the intact lower limb or the residual limb that significantly impacted or limited gait or joint range of motion. Participant demographic information including etiology and years of prosthesis use is described in Table 1. In this study, instead of using their respective passive prostheses, the experiment was conducted using the experimental powered knee prosthesis with bimodal stumble recovery responses. The experimental protocol was approved by the Vanderbilt University Institutional Review Board. All participants gave their written informed consent. The experimental setup for both studies consisted of walking on a split-belt, force-instrumented treadmill (Bertec, Columbus, USA) at 0.8 m/s while wearing a force-instrumented, full-body harness. The walking speed was selected to replicate previous stumble recovery studies with transfemoral prosthesis users (Shirota et al., 2015), while also allowing for a comfortable walking speed for the entire cohort based on pilot testing and prior experiments. Notably, the able-bodied control data used in this work was collected using the same experimental protocol except at 1.1 m/s, similarly, to replicate previous studies (Schillings et al., 2000) and to provide a comfortable walking speed across the cohort. While the difference in walking speed between the prosthesis user group and the control group potentially limits comparisons between the cohorts, the comparison may still provide useful insights. Additionally, preliminary data suggests that the able-bodied control stumble recovery response may not differ significantly between 0.8 m/s and 1.1 m/s (Eveld et al., 2021).

**TABLE 1:** Participant demographic information.

Participant	Age	Sex	Prosthetic side	Etiology	Years of prosthesis use	Prescribed prosthesis
Participant 1	32	Male	Left	Trauma	4	Blatchford KX06
Participant 2	28	Female	Right	Congenital	27	Ottobock C-leg 4
Participant 3	62	Male	Right	Trauma	49	Ottobock C-leg 4

Stumble perturbations were introduced after a randomized number of strides. An in-house stumble perturbation system was used to induce obstacle perturbations. The system consisted of a pair of ramps at the front of the treadmill that deposited 16 kg (35 lbs) steel blocks that measured 20 cm wide, 12.5 cm long, and 7.5 cm high (8.125″ x 5″ x 3″) onto the treadmill at specific times to target selected points in swing phase using a predictive targeting algorithm. More on the perturbation system design and validation can be found in (King et al., 2019).

During the experiment, participants wore sensory occlusion equipment to further prevent anticipation of the perturbations. Sensory occlusion equipment included white noise earbuds and passive noise canceling headphones to block sound as well as dribble goggles to block the inferior visual field. Participants also performed Serial Sevens (i.e., counting backwards by sevens from a randomized initial seed number) to act as a distraction task and to prevent compensatory behavior. Participants also received visual feedback in the form of arrows on a screen in front of them to keep them centered on the treadmill. Perturbations were applied to both the prosthesis side and the sound side after a randomized number of strides to further prevent anticipation. Perturbations were applied at early, mid and late swing phase with each participant experiencing at least three perturbations on their passive prosthesis and at least 13 on the powered prosthesis. Proportionally more early swing perturbations were applied during the powered prosthesis experiment to capture more data on the elevating response. Infrared motion capture (Vicon, Oxford, GBR) was used for kinematic data collection with a full body motion capture marker set including feet, shank, thigh, pelvis, torso, upper arm, and forearm segments. The handrails were removed from the treadmill to prevent them from being used for assistance and altering the recovery. However, a full-body, load cell-instrumented harness was worn both to protect the participants from contacting the treadmill during a fall as well to measure the amount of bodyweight assistance the harness provided during each trial.

Prior to data collection on the powered prosthesis, an acclimation period was provided to allow the participants to adapt to the powered prosthesis and experience the novel recovery strategies, particularly the elevating strategy since it is generally not an accessible stumble recovery response for prosthesis users when using passive prostheses. During the acclimation period, participants walked with the powered prosthesis and experienced the response of the prosthesis at different perturbation timings without sensory occlusion equipment and with the handrails present. As they became comfortable with the response, more elements of the experimental protocol were gradually included starting with each piece of sensory occlusion equipment, followed by the removal of one handrail, and finally the removal of both handrails. The acclimation period ended once each participant stated that they were comfortable with the powered prosthesis controller, and they were observed responding to similarly timed perturbations in a consistent manner (using the same response, but not inherently a recovery, for at least three perturbations in a row). The acclimation period typically consisted of one to three 2-h sessions. Participant 1 acclimated the quickest with only one session, followed by Participant 3 with two sessions, and Participant 2 with three sessions.

### 2.3 Data processing and analysis

Motion capture marker data were preprocessed in Vicon Nexus (Vicon, Oxford, GBR) to label and gap fill the data. Gap filling was performed using a pattern fill based on markers from the same segment, unless the gap was five frames or less in which case a spline fill was used. Joint-level kinematic and kinetic data were computed using Visual 3D inverse dynamics software (C-Motion, Germantown, USA) and analyzed in MATLAB (Mathworks, Natick, USA). Force plate and motion capture data were filtered with a zero-phase, third order, low-pass Butterworth filter with a cut-off frequency of 15 and 6 Hz, respectively. Perturbation timing was determined using a spike in the anterior-posterior force plate data at the time of the perturbation. Falls were defined as ≥50% bodyweight seconds of harness assistance (i.e., the integral of the measured load through the harness normalized to the participant’s bodyweight), while a harness assisted recovery was defined as ≥10% bodyweight seconds of harness assistance while remaining <50% bodyweight seconds. Bodyweight seconds has been used in previous studies as a metric to contextualize the harness assistance impulse during stumble recovery (Crenshaw et al., 2013b). Falls and harness assists are collectively referred to as failed recoveries. In the event of force plate crossover steps following the perturbation, gait events were determined using vertical foot CoM velocity (O’Connor et al., 2007). Rather than discarding crossover trials, the kinematic gait event determination method was used to preserve as much data and participant effort as possible due to the physically challenging and time-consuming nature of stumble perturbation trials limiting the maximum number of trials participants were willing or able to complete in a single session.

In order to avoid a reliance entirely on discrete, fall-based outcomes, key biomechanical recovery outcome metrics are reported to describe the quality of the recovery in a continuous manner. Trunk metrics including peak trunk angle and peak trunk angular velocity during the recovery, defined as the first two strides following the perturbation, relative to the value at perturbation are reported as indicators of quality of recovery, as improved torso control, and therefore reduced CoM displacement, indicate reduced likelihood of falling (Pavol et al., 2001; Crenshaw et al., 2012). Additionally, forward reach, defined as the distance from body CoM to tripped foot CoM at first ground contact after the perturbation, is also reported as an indicator of recovery quality since as greater forward reach indicates that the swing limb is extended further ahead of the individual prior to ground contact, increasing their base of support size. Alternatively, reduced or negative forward reach indicates a shrinking base of support, limiting the time the individual has to avoid a fall. Forward reach is relative to the value at perturbation and normalized to participant height. In previous work, trunk metrics in early swing and forward reach across all portions of swing phase were notably worse for prosthesis users compared to able-bodied controls (King et al., 2024). Additionally, knee angle at first ground contact and peak thigh abduction with respect to the value at perturbation are reported to provide further kinematic characterization of the recovery. Knee angle at ground contact is an important metric for passive prostheses as it has been reported that >30° of knee flexion increases the likelihood of knee buckling (Crenshaw et al., 2013b). Thigh abduction is an analog for hip circumduction, which is used as compensatory response to account for the inability to flex the prosthetic knee joint adequately to cross over the obstacle during a recovery.

## 3 Results

Results are separated into three segments of swing phase: early (<35%) , mid (35−54%) and late (>54%) . Using able-bodied recovery strategies for context, early swing typically corresponds to the use of the elevating recovery strategy; late swing to the lowering strategy; and mid swing to the elevating or delayed lowering strategies (Eng et al., 1994; Schillings et al., 2000; King et al., 2019; Eveld et al., 2021). Each participant with each prosthesis (i.e., powered and prescribed, passive) has at least one perturbation in each category, with the exception of Participant 2, who did not incur a mid-swing perturbation with her passive prosthesis due to experimental targeting error. The data herein is presented as a case series without statistical analysis due to the low number of samples preventing reliable results for many of the passive prosthesis conditions. The interquartile range (IQR) from the able-bodied control cohort is included for relevant metrics. Able-bodied harness assistance impulse is excluded due to effectively no harness utilization by the able-bodied cohort. Additionally, able-bodied knee angle data is excluded as the knee angle metric is solely relevant to transfemoral prosthesis users due to the lack of direct control of the joint. In the supplemental materials, a video is included with representative trials for each participant using both the powered prosthesis and their passive prosthesis in early and late swing phase. Note, in order to maintain a consistent orientation in the direction of walking in the video, Participant 1’s video has been flipped.

### 3.1 Overview

**FIGURE 2:**
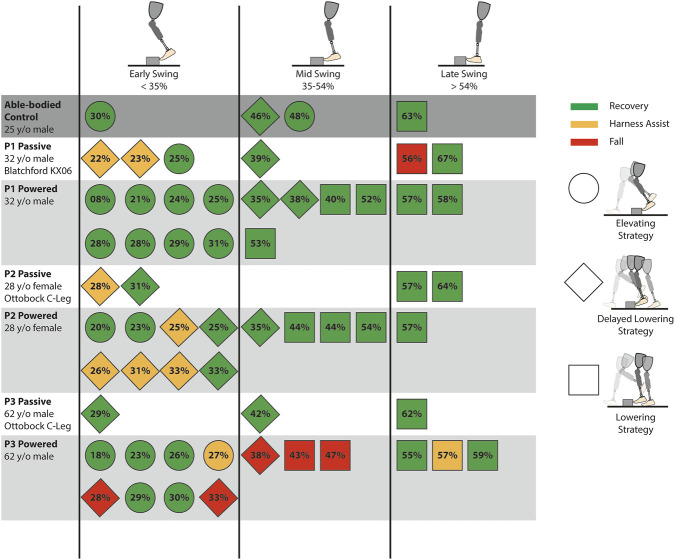
Recovery outcomes across swing phase for each participant. White rows are passive prosthesis outcomes and grey rows are powered prosthesis outcomes. Green indicates a recovery, yellow indicates a harness assist, and red indicates a fall. Elevating strategies are depicted by circles, delayed lowering strategies are depicted by diamonds, and lowering strategies are depicted by squares. The percentage of swing phase of each perturbation is indicated in the shape.

A summary of recovery outcomes and strategies is provided in Figure 2. As reported in previous work, early swing proved to be the most difficult period to recover from perturbations for prosthesis users (King et al., 2024). For the passive prostheses, the delayed lowering and lowering strategies generally dominated, with only one instance of the elevating strategy in early swing by Participant 1. However, the powered prosthesis allowed all three participants to utilize the elevating strategy in early swing, two of whom (Participant 1 and 3) demonstrated consistent, repeated use. Two participants (Participant 2 and 3) experienced intent mismatch with the powered prosthesis and still used the delayed lowering strategy at some point in early swing phase, abandoning the elevating strategy after the powered prosthesis selected it. Participant 2 used the delayed lowering strategy more often than the elevating strategy in early swing, likely due to the fact that congenital limb difference would have prevented her from ever using the elevating strategy prior to this experiment. In mid and late swing for both prosthesis classes, participants converged on using the delayed lowering and lowering strategies.

Comparing the powered prosthesis to the passive prostheses across all of swing phase, harness assistance-based recovery outcomes show that Participant 1 (P1) recovered from 100% of perturbations with the powered prosthesis versus 50% with the passive prosthesis; Participant 2 (P2) recovered from 69% of perturbations with the powered prosthesis versus 75% with the passive prosthesis; and Participant 3 (P3) recovered from 50% of perturbations with the powered prosthesis versus 100% with the passive prosthesis. Of the three participants on their passive prostheses, Participant 3’s general recovery strategy was the most unorthodox and utilized rotating his body perpendicular to the direction of the treadmill’s motion, keeping the knee joint out of the sagittal plane to prevent it from buckling while side stepping for several strides until he regained his balance. While the addition of whole-body rotation to the recovery response worked well for avoiding falls and reducing trunk displacement, it greatly increased his time to return to his unperturbed walking pattern.

### 3.2 Early swing

**FIGURE 3:**
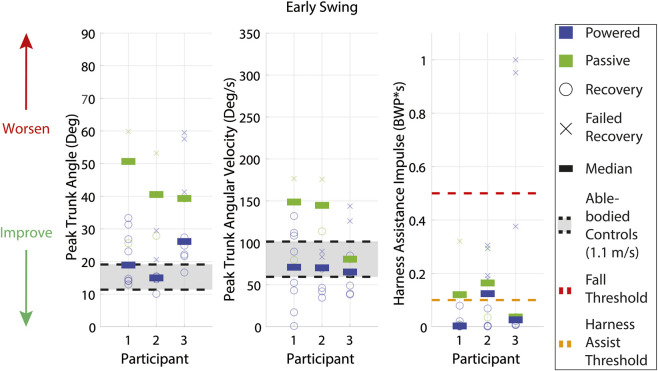
Early swing trunk and harness assistance recovery metrics. Left is peak trunk angle during the recovery relative to the value at perturbation, middle is peak trunk angular velocity during the recovery relative to the value at perturbation, and right is maximum harness assistance impulse during the recovery. Powered trials are represented in blue while Passive trials are represented in green. Empty circles are recoveries, X’s are failed recoveries, and the bars are the medians for each prosthesis. The grey region bordered with dashed black lines is the interquartile range for each metric from the able-bodied controls at 1.1 m/s. Able-bodied control data are not included for harness assistance due to essentially no harness usage. The yellow dashed line is the harness assist threshold and the red dashed line is the fall threshold.

Trunk and harness assistance metrics for early swing are shown in Figure 3. The powered prosthesis improved trunk metric outcomes for all three participants compared to their passive prostheses (Trunk Angle Median: **P1**- Passive: 50.6° , Powered: 18.9° , **P2**- Passive: 40.5° , Powered: 15.0° , **P3**- Passive: 39.3° , Powered: 26.1° , Trunk Angular Velocity Median: **P1**- Passive: 148.7°/s , Powered: 70.9°/s , **P2**- Passive: 144.7°/s , Powered: 70.2°/s , **P3**- Passive: 80.5°/s , Powered: 64.2°/s ). Participants 1 and 2 demonstrated the largest reduction in both median peak trunk angle and median peak trunk angular velocity, while Participant 3 had only a small reduction. However, Participant 3 turned out of the plane of motion of the treadmill during his recovery with his passive prosthesis and stayed in this orientation for several strides after, which reduced the total amount of trunk deflection while increasing his total number of strides to return to steady state walking. On the powered prosthesis, Participant 3 was generally able to recover in a single stride and had reduced trunk displacement without the need to turn sideways. Additionally, the powered prosthesis brought Participants 1 and 2 into the range of the able-bodied controls’ (AB) trunk metrics at 1.1 m/s (**AB** Trunk Angle IQR: 19.1° - 11.4° , **AB** Trunk Angular Velocity IQR: 101.5°/s - 59.5°/s ). For Participant 1, the powered prosthesis reduced harness assistance during recovery (**P1**- Passive: 12.0%, Powered: 0.3%) while Participant 2 had a slight decrease (**P2**- Passive: 16.4%, Powered: 12.4%) and Participant 3 maintained similar median harness assistance across both prostheses (**P3**- Passive: 3.6%, Powered: 2.6%).

**FIGURE 4:**
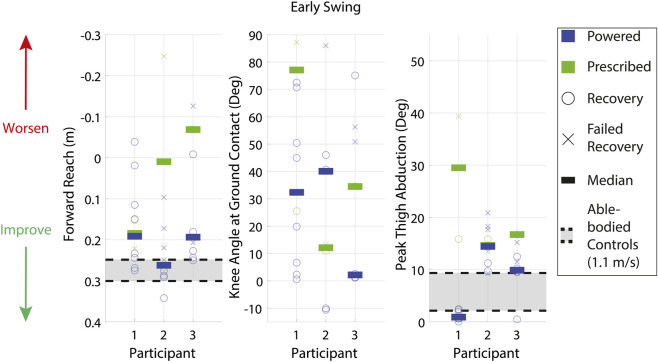
Early swing step and lower limb recovery metrics. Left is forward reach (distance from body CoM to tripped foot CoM) at the end of the recovery stride relative to the value at perturbation and normalized to participant height, middle is knee angle at first ground contact following the perturbation, and right is peak thigh abduction during the recovery relative to the value at perturbation. Forward reach has an inverted vertical axis to maintain the visual trend across the plots. Positive forward reach indicates the swing limb has been extended with respect to the body CoM, increasing the base of support, whereas negative forward reach indicates the limb has been retracted, reducing the base of support. Powered trials are represented in blue while Passive trials are represented in green. Empty circles are recoveries, X’s are failed recoveries, and the bars are the medians for each prosthesis. The grey region bordered with dashed black lines is the interquartile range for each metric from the able-bodied controls at 1.1 m/s. Able-bodied control data are not included for knee angle since it does not correlate to quality of recovery in that population.

Early swing step and lower limb metrics are shown in Figure 4. Forward reach was improved for Participants 2 and 3 (**P2**- Passive: 0.00 m, Powered: 0.26 m, **P3**- Passive: −0.07 m, Powered: 0.19 m), while Participant 1 maintained similar forward reach (**P1**- Passive: 0.18 m, Powered: 0.19). Participant 2’s forward reach with the powered prosthesis was in the range of the able-bodied controls (**AB** IQR: 0.30 m–0.25 m). Knee angle at first ground contact was reduced for Participants 1 and 3 (**P1**- Passive: 77.1° , Powered: 32.4° , **P3**- Passive: 34.5° , Powered: 1.2° ), and although Participant 2 had an increase in median knee flexion (**P2**- Passive: 11.0° , Powered: 40.1° ), no recoveries with the powered prosthesis resulted in the knee buckling. Participants 1 and 3 also saw a reduction in thigh abduction (i.e., hip circumduction) during recovery when using the powered prosthesis (**P1**- Passive: 29.5° , Powered: 0.7° , **P3**- Passive: 16.7° , Powered: 9.9° ), while Participant 2 maintained similar thigh abduction (**P2**- Passive: 14.6° , Powered: 14.5° ).

### 3.3 Mid swing

**FIGURE 5:**
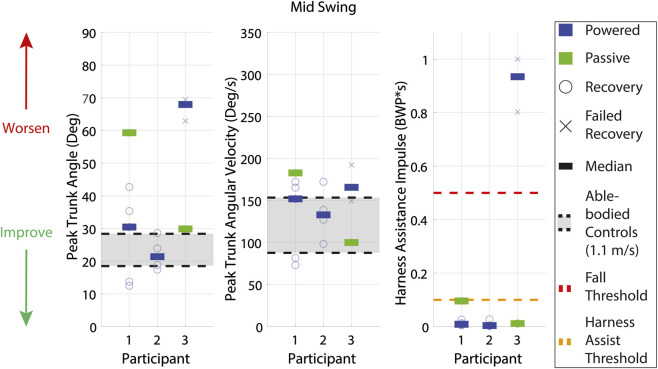
Mid swing trunk and harness assistance recovery metrics. Left is peak trunk angle during the recovery relative to the value at perturbation, middle is peak trunk angular velocity during the recovery relative to the value at perturbation, and right is maximum harness assistance impulse during the recovery. Powered trials are represented in blue while Passive trials are represented in green. Empty circles are recoveries, X’s are failed recoveries, and the bars are the medians for each prosthesis. The grey region bordered with dashed black lines is the interquartile range for each metric from the able-bodied controls at 1.1 m/s. Able-bodied control data are not included for harness assistance due to essentially no harness usage. The yellow dashed line is the harness assist threshold and the red dashed line is the fall threshold.

Trunk and harness assistance metrics for mid swing are shown in Figure 5. In mid swing, the powered prosthesis improved trunk metric outcomes for Participant 1 (Trunk Angle Median: **P1**- Passive: 59.3° , Powered: 30.5° , Trunk Angular Velocity Median: **P1**- Passive: 182.9°/s , Powered: 152.0°/s ). Participant 2 did not experience any mid swing perturbations on her passive prosthesis, however, her trunk metrics with the powered prosthesis were similar in magnitude to Participant 1 (Trunk Angle Median: **P2**- Powered: 21.4° , Trunk Angular Velocity Median: **P2**- Powered: 133°/s ) and within the able-bodied range (**AB** Trunk Angle IQR: 28.4° - 18.5° , **AB** Trunk Angular Velocity IQR: 153.5°/s - 87.7°/s ). Participant 3 experienced an increase in his trunk metrics with the powered prosthesis as he fell every time in mid swing (Trunk Angle Median: **P3**- Passive: 29.9° , Powered: 68.0° , Trunk Angular Velocity Median: **P3**- Passive: 100.0°/s , Powered: 165.7°/s ). Due to attempting to use the lowering strategy without turning sideways while using the powered prosthesis, he struggled with mid swing perturbations the most since the elevating strategy was generally not observed in this region of swing and he was unable to successfully perform the traditional lowering strategy. For harness assistance, Participant 1 showed a reduction (**P1**- Passive: 9.6%, Powered: 0.9%), Participant 2 showed similar levels to Participant 1 (**P2**- Powered: 0.4%), and Participant 3 showed an increase (**P3**- Passive: 1.1%, Powered: 93.5%).

**FIGURE 6:**
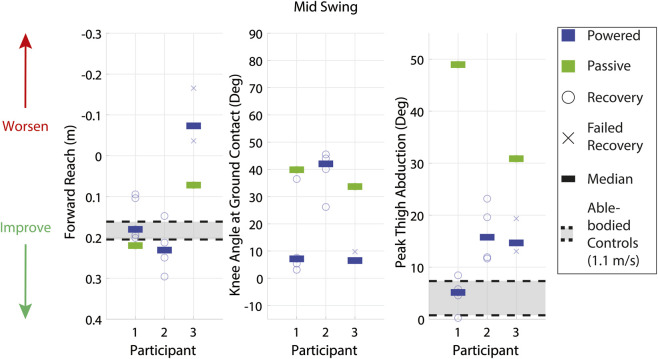
Mid swing step and lower limb recovery metrics. Left is forward reach (distance from body CoM to tripped foot CoM) at the end of the recovery stride relative to the value at perturbation and normalized to participant height, middle is knee angle at first ground contact following the perturbation, and right is peak thigh abduction during the recovery relative to the value at perturbation. Forward reach has an inverted vertical axis to maintain the visual trend across the plots. Positive forward reach indicates the swing limb has been extended with respect to the body CoM, increasing the base of support, whereas negative forward reach indicates the limb has been retracted, reducing the base of support. Powered trials are represented in blue while Passive trials are represented in green. Empty circles are recoveries, X’s are failed recoveries, and the bars are the medians for each prosthesis. The grey region bordered with dashed black lines is the interquartile range for each metric from the able-bodied controls at 1.1 m/s. Able-bodied control data are not included for knee angle since it does not correlate to quality of recovery in that population.

Mid swing step and lower limb recovery metrics are shown in Figure 6. Forward reach was similar for Participant 1 across both prostheses (**P1**- Passive: 0.22 m, Powered: 0.18 m), and Participant 2 was similar to Participant 1 (**P2**- Powered: 0.23 m). Participant 3 showed a decrease in forward reach (**P3**- Passive: 0.07 m, Powered: −0.07 m). Participant 2 on the powered prosthesis and Participant 1 on both the passive and powered prostheses performed as well or better than the able-bodied control range for forward reach (**AB** IQR: 0.21 m–0.16 m). Knee angle at first ground contact was reduced for Participants 1 and 3 when using the powered prosthesis (**P1**- Passive: 39.9° , Powered: 7.1° , **P3**- Passive: 33.7° , Powered: 6.5° ), while Participant 2 experienced higher knee flexion (**P2**- Powered: 42.0° ), although no knee buckling occurred with the powered prosthesis. Participant 1 demonstrated a large reduction in thigh abduction with the powered prosthesis compared to his passive prosthesis (**P1**- Passive: 49.0° , Powered: 5.1° ) and was within the able-bodied control range (**AB** IQR: 7.3° - 0.7° ). Participant 3 also demonstrated a reduction (**P3**- Passive: 30.9° , Powered: 14.7° ) but was still outside the control range. Participant 2 demonstrated similar thigh abduction to Participant 3 (**P2**- Powered: 15.8° ).

### 3.4 Late swing

**FIGURE 7:**
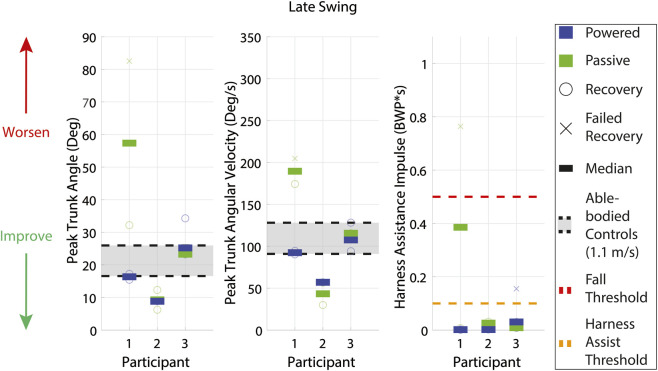
Late swing trunk and harness assistance recovery metrics. Left is peak trunk angle during the recovery relative to the value at perturbation, middle is peak trunk angular velocity during the recovery relative to the value at perturbation, and right is maximum harness assistance impulse during the recovery. Powered trials are represented in blue while Passive trials are represented in green. Empty circles are recoveries, X’s are failed recoveries, and the bars are the medians for each prosthesis. The grey region bordered with dashed black lines is the interquartile range for each metric from the able-bodied controls at 1.1 m/s. Able-bodied control data are not included for harness assistance due to essentially no harness usage. The yellow dashed line is the harness assist threshold and the red dashed line is the fall threshold.

Late swing trunk and harness assistance metrics are shown in Figure 7. In late swing, Participant 1 demonstrated a notable reduction in peak trunk angle (**P1**- Passive: 57.4° , Powered: 16.4° ) and trunk angular velocity (**P1**- Passive: 189.5°/s , Powered: 94.4°/s ) with the powered prosthesis while Participants 2 and 3 had similar trunk metrics with both prostheses (Trunk Angle Median: **P2**- Passive: 9.3° , Powered: 8.8° , **P3**- Passive: 23.3° , Powered: 25.2° , Trunk Angular Velocity Median: **P2**- Passive: 43.3°/s , Powered: 57.1°/s , **P3**- Passive: 115.7°/s , Powered: 107.5°/s ). Participant 1’s trunk metrics were within or even below the able-bodied control range (**AB** Trunk Angle IQR: 26.0° - 16.6° , **AB** Trunk Angular Velocity IQR: 128.1°/s - 90.9°/s ) with the powered prosthesis and Participants 2 and 3 were within or below the able-bodied range for both prostheses. Harness assistance was reduced for Participant 1 (**P1**- Passive: 38.6%, Powered: 0.8%) and generally maintained for Participant 2 and Participant 3 (**P2**- Passive: 2.6%, Powered: 0.2%, **P3**- Passive: 0.9%, Powered: 3.0%).

**FIGURE 8:**
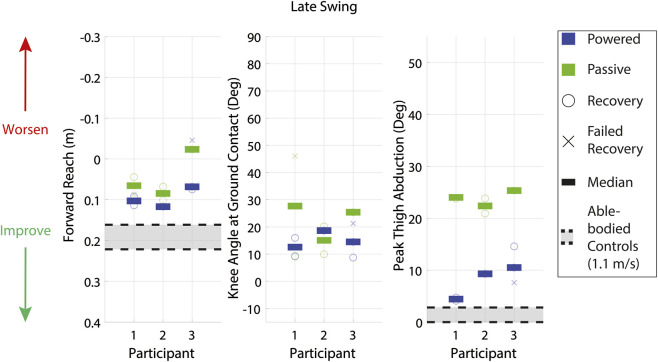
Late swing step and lower limb recovery metrics. Left is forward reach (distance from body CoM to tripped foot CoM) at the end of the recovery stride relative to the value at perturbation and normalized to participant height, middle is knee angle at first ground contact following the perturbation, and right is peak thigh abduction during the recovery relative to the value at perturbation. Forward reach has an inverted vertical axis to maintain the visual trend across the plots. Positive forward reach indicates the swing limb has been extended with respect to the body CoM, increasing the base of support, whereas negative forward reach indicates the limb has been retracted, reducing the base of support. Powered trials are represented in blue while Passive trials are represented in green. Empty circles are recoveries, X’s are failed recoveries, and the bars are the medians for each prosthesis. The grey region bordered with dashed black lines is the interquartile range for each metric from the able-bodied controls at 1.1 m/s. Able-bodied control data are not included for knee angle since it does not correlate to quality of recovery in that population.

Late swing step and lower limb recovery metrics are shown in Figure 8. Forward reach is slightly increased for all three participants (**P1**- Passive: 0.07 m, Powered: 0.10 m, **P2**- Passive: 0.08 m, Powered: 0.12 m, **P3**- Passive: −0.02 m, Powered: 0.07 m), but not to the able-bodied control range (**AB** IQR: 0.22 m–0.16 m). Knee angle at ground contact was reduced for Participants 1 and 3 (**P1**- Passive: 27.7° , Powered: 12.6° , **P3**- Passive: 25.4° , Powered: 14.5° ), and no knee buckling was observed, even for Participant 2 (**P2**- Passive: 15.1° , Powered: 18.7° ). Thigh abduction was notably decreased for all three participants (**P1**- Passive: 23.8° , Powered: 4.4° , **P2**- Passive: 22.4° , Powered: 9.3° , **P3**- Passive: 25.3° , Powered: 10.5° ).

## 4 Discussion

Based on the recovery metrics, the powered knee prosthesis with the inclusion of powered stumble recovery responses addressed several key deficits observed in the prescribed, passive prostheses in this preliminary evaluation. While participants did not reduce their fall or failed recovery rate by using the powered prosthesis, the improvement in several key recovery metrics demonstrates promise for elements of the powered prosthesis’s behavior and merits further exploration. First, the robust stance support provided by the powered prosthesis fully eliminated knee buckling. Second, improved swing extension assistance ensured that the powered prosthesis was ready for load acceptance at heel strike (Crenshaw et al., 2013b). Two out of three participants (Participants 1 and 3) showed a consistent reduction in knee flexion angle at first ground contact across all of swing phase. While Participant 2 did not show improved knee angle at ground contact, she still did not experience knee buckling. However, she struggled with consistent use of the elevating strategy more than other participants in the cohort, often using a delayed lowering response which suggests difficulty coordinating with the powered prosthesis stumble recovery controller. The poor coordination with the powered prosthesis caused her to bring her foot to the ground ahead of full knee extension in the manner of a delayed lowering strategy. The intent mismatch between her and the prosthesis may, in part, be due to having congenital limb difference, and therefore she has likely never used the elevating strategy on her affected limb prior to this study. Third, the swing flexion initiation assistance resulted in a reduction in thigh abduction for all three participants across all of swing phase except for Participant 2 in early swing phase where thigh abduction was maintained. The decrease in thigh abduction (i.e., hip circumduction) indicates that the knee flexion assistance allowed users to initiate swing and cross over the obstacle rather than circumducting around it. Lastly, the use of the elevating strategy in early swing reduced peak trunk angle and angular velocity in all participants, improved harness assistance for Participant 1 (maintained for Participants 2 and 3), and improved forward reach for Participants 2 and 3 (maintained for Participant 1). The majority of the recovery outcome metrics were improved for all participants when using the powered prosthesis, indicating improved CoM control and generally improved base of support adjustment. While Participant 2 struggled to use the elevating strategy consistently, frequently abandoning the response part way through and using the delayed lowering strategy instead, she still demonstrated a reduction in the median of both trunk metrics and forward reach with maintained harness assistance.

Interestingly, the frequency of intent mismatches (i.e., abandoning the elevating strategy in favor of the delayed lowering strategy) followed a consistent inverse trend with the number of acclimation sessions for each participant. Participant 2 experienced the most intent mismatches while undergoing three acclimation sessions, Participant 3 experienced the second most intent mismatches with two acclimation sessions, and Participant 1 had no intent mismatches with one acclimation session. The trend in intent mismatches is also consistent with the amount of time each participant has been using a prosthesis (entire life (27 years) for Participant 2, 49 years for Participant 3, and 4 years for Participant 1). The time of prosthesis use may be analogous to the amount of time since they have consistently used the elevating strategy. The trends in intent mismatch incidence suggest that challenges with acclimation may be related to the time since using the elevating strategy and that these challenges persisted beyond the acclimation period even with the selected acclimation criteria, highlighting the need for further study of the acclimation response itself.

Improvements to trunk and forward reach metrics with the powered prosthesis were seen in mid and late swing as well. Participant 1 demonstrated improved trunk and harness assistance metrics in mid and late swing with maintained forward reach. Participant 2 demonstrated maintained metrics in late swing (no comparison case in mid swing). Participant 3 was the only participant to demonstrate worse recovery outcome metrics in any portion of swing phase with worse trunk, harness assistance, and forward reach metrics in mid swing. However, Participant 3 did demonstrate improved forward reach in late swing along with maintained trunk and harness assistance metrics. The results indicate that throughout mid to late swing, use of the powered prosthesis resulted in improved or maintained CoM control and base of support adjustments except for Participant 3 in mid swing.

Additionally, when using the powered prosthesis, many of the recovery metrics were frequently within the able-bodied controls’ recovery metric IQRs, though it is important to note the difference in walking speed between the two cohorts as well as the limited number of samples for the prosthesis user cohort. Participant 1’s and Participant 2’s trunk metrics were generally comparable to the able-bodied controls across swing phase while using the powered prosthesis. Forward reach was improved to the range of the able-bodied controls for Participant 1 in mid swing and Participant 2 in early and mid swing. Thigh abduction was also improved to the range of able-bodied controls for Participant 1 in early and mid swing and Participant 3 in early swing. Previous work demonstrated early swing was where prosthesis users struggled the most with deficits in both trunk and forward reach metrics compared to able-bodied controls (King et al., 2024). In the current study in late swing, forward reach and thigh abduction were still somewhat deficient, however.

Throughout swing phase, the failed recoveries with the powered prosthesis were often associated with a lack of step initiation following the perturbation and poor base of support adjustment, suggesting the need for further improvement in swing initiation assistance and training. Participant 1 being the only exception, as he experienced no failed recoveries with the powered prosthesis and maintained robust step initiation and base of support adjustments. However, with his passive prosthesis he experienced several failed recoveries due to delayed step initiation and poor base of support adjustments as well as knee buckling. Participant 2’s powered prosthesis failed recoveries occurred during intent mismatch delayed lowering responses where she remained caught on the obstacle after the perturbation due to delayed step initiation, and at which point she would resort to hopping on her sound limb. Her failed recovery on her passive prosthesis closely mirrored the failed recoveries on the powered prosthesis with remaining caught on the obstacle and failing to initiate the next step. While Participant 3 experienced no failed recoveries with his passive prosthesis, facilitated by turning his body out of the plane of motion of the treadmill to avoid knee buckling, he struggled with failed recoveries on the powered prosthesis primarily in mid swing phase. Most of his failed recoveries were during delayed lowering and lowering responses where a combination of delayed step initiation, poor base of support adjustment, and poor trunk control resulted in the failed recoveries. For his one elevating strategy failed recovery, he successfully cleared the obstacle using the elevating strategy but fell several strides later due to a loss of balance. The failed recovery outcomes with the powered prosthesis suggest that while stance support and swing assistance have improved recovery outcomes, swing initiation assistance is not enough on its own and is primarily associated with the failed recoveries in addition to intent mismatches.

The recovery metric outcomes from this preliminary work suggest that the inclusion of powered elevating and lowering strategies in a powered knee prosthesis may improve an individual’s ability to recover from obstacle perturbations better than current commercially-available, passive prostheses. Additionally, the powered prosthesis may improve key recovery metrics related to CoM control and base of support adjustment to a level comparable with the range of able-bodied controls, though the small sample size and limited repeated measures prevent conclusive, direct comparisons between the two groups. However, such improvements could potentially play a role in mitigating the risk of real-world falls and subsequent injury in the high-risk transfemoral prosthesis user population with further study. Additionally, the increased harness assistance during the use of the powered prosthesis suggests the need for further investigation to increase coordination between the user and the prosthesis by avoiding recovery strategy intent mismatches between the two. In part, with the inclusion of more data demonstrating how people react to different perturbations across swing phase, the powered stumble recovery decision algorithm can be further refined, or possibly personalized, to increase the coordination between the prosthesis and the user and to provide adaptive behaviors that adjust to the user’s performance over time to better facilitate acclimation. As such, further study refining the acclimation procedure is also critical to improve users’ utilization of the elevating strategy as well as overall coordination with the powered stumble recovery behavior. Even with the attempts to standardize the acclimation period in this study, the relative lack of familiarity with the powered prosthesis compared to the passive prostheses likely played a role in the variable responses from the participants. In juxtaposition to their daily, extended use of their passive prosthesis, they only used the powered prosthesis in a limited laboratory setting for a few hours at a time across a limited number of sessions. Therefore, depending on how comfortable an individual is with using a new prosthesis, people may demonstrate different acclimation rates and different levels of confidence with the powered prosthesis. Even with the acclimation period in this study, participants very likely still had greater confidence when using their prescribed, passive prosthesis.

A significant limitation of the current study is the limited, unbalanced data points across the regions of swing, participants, and prosthesis types. Continued testing with an increased cohort size with a greater number of perturbations per participant would improve the understanding of the potential benefit of the intervention and address a key limitation of the current study. Additionally, matching the walking speed velocity between the prosthesis user and able-bodied control cohorts is an important adjustment to improve the quality of the comparison between the groups. Though preliminary evidence suggests that the stumble recovery response for healthy, young, able-bodied adults may not vary significantly between the walking speeds used in this study (Eveld et al., 2021). Use of an ABA design in the experimental protocol would also likely improve the quality of the data, although participants may have limited tolerance for extensive, repeated stumble perturbations. An extended acclimation protocol with the powered prosthesis would also likely improve the experimental outcomes, particularly one in which participants can take the prosthesis home and acclimate over a longer period of time prior to testing. Further, a longitudinal study across the acclimation period would improve the understanding of the time required to reach steady state acclimation and how the benefit evolves and plateaus over time. Implementing stumble recovery behaviors on a lower impedance, semi-powered prosthesis (Lee et al., 2019; Lenzi et al., 2018; Culver et al., 2022; Bartlett et al., 2022) may make the powered stumble recovery responses easier to use and provide further benefit, as the lower impedance profile would make the prosthesis easier for the user to manipulate directly with their hip on their affected side for improved swing control. The lower knee impedance would allow for true ballistic motion at the knee during regular walking rather than emulation as is the case with the trajectory control in the powered prosthesis used in this study. Additionally, reduced force transmission through the lower impedance, semi-powered prosthesis during a perturbation may further reduce undesirable effects such as excessive trunk deflection. The lower impedance profile may also improve the acclimation rate since the mechanics of the prosthesis may be more similar to the passive prostheses with which participants are already familiar. Lastly, for real world implementation and success, the powered prosthesis stumble recovery behaviors must be integrated with additional functionality such as preemptive obstacle detection (Lawson et al., 2010; Zhang et al., 2011), obstacle avoidance (Thatte et al., 2019; Gordon et al., 2019; Mendez et al., 2020; Chen et al., 2024; Cheng et al., 2023; Rezazadeh et al., 2019; Zhu et al., 2025), and general perturbation rejection behaviors (Thatte and Geyer, 2015). Additionally, coupling the use of such a powered prosthesis with task-specific stumble perturbation gait training may further bolster the real world benefit to the user, much like when training is performed with passive prostheses (Crenshaw et al., 2013a).

Overall, the reduction of the four primary functional deficits as well as the improvement to both trunk metrics and lower limb step metrics across this cohort suggests the potential for the benefit of powered prosthesis stumble recovery responses. The powered prosthesis’s benefits include improved stance support and swing assistance as well as the reintroduction of the elevating strategy for transfemoral prosthesis users. Further integration of such behaviors into commercial prosthesis designs may improve stumble recovery outcomes for transfemoral prosthesis users and has the potential to reduce the risk of falls and related injuries.

## 5 Conclusion

This paper examines the prospective value of employing bimodal powered stumble recovery responses in a powered knee prosthesis. The paper specifically compares the stumble recovery response of transfemoral prosthesis users across swing phase with their prescribed, passive prostheses relative to a powered prosthesis with improved stance support, improved swing assistance, and a bimodal stumble recovery response that includes both an elevating and a lowering strategy. The powered prosthesis intervention did not change the rate of successful or failed recoveries, as defined in this study; however, the powered prosthesis did improve an array of stumble recovery metrics known in literature to correlate to stumble perturbation severity. Specifically, trunk control and recovery step metrics improved, particularly in early swing. Recovery metrics were largely improved or maintained across the study’s cohort, even demonstrating improvements to a range comparable to able-bodied controls at a faster walking speed. Only one participant demonstrated consistently worse performance metrics in one portion of swing phase (Participant 3 in mid swing). Since this study was performed as a case series, further exploration of the prospective benefit of this intervention would be improved with a larger cohort, more repeated measures, and an extended acclimation period. In this preliminary investigation, the powered prosthesis demonstrated promise for improving the recovery outcomes of transfemoral prostheses users.

## Data Availability

The raw data supporting the conclusions of this article will be made available by the authors, without undue reservation.

## References

[B1] Afschrift M. Van Asseldonk E. Van Mierlo M. Van Der Kooij H. De Groote F. (2024). “Assisting global balance recovery responses during perturbed walking with ankle exoskeletons,” in 2024 10th IEEE RAS/EMBS International Conference for Biomedical Robotics and Biomechatronics (Biorob) (IEEE), 508–513.

[B2] Bartlett H. L. King S. T. Goldfarb M. Lawson B. E. (2022). Design and assist-as-needed control of a lightly powered prosthetic knee. IEEE Trans. Med. Robotics Bionics 4, 490–501. 10.1109/tmrb.2022.3161068

[B3] Blumentritt S. Schmalz T. Jarasch R. (2009). The safety of c-leg: biomechanical tests. JPO J. Prosthetics Orthot. 21, 2–15. 10.1097/jpo.0b013e318192e96a

[B4] Chen C. Chen X. Yin S. Wang Y. Huang B. Leng Y. (2024). “Enhancing prosthetic safety and environmental adaptability: a visual-inertial prosthesis motion estimation approach on uneven terrains,” in 2024 IEEE/RSJ International Conference on Intelligent Robots and Systems (IROS) (IEEE), 206–213.

[B5] Cheng S. Laubscher C. A. Gregg R. D. (2023). Automatic stub avoidance for a powered prosthetic leg over stairs and obstacles. IEEE Trans. Biomed. Eng. 71, 1499–1510. 10.1109/TBME.2023.3340628 38060364 PMC11035099

[B6] Crenshaw J. R. Rosenblatt N. J. Hurt C. P. Grabiner M. D. (2012). The discriminant capabilities of stability measures, trunk kinematics, and step kinematics in classifying successful and failed compensatory stepping responses by young adults. J. Biomechanics 45, 129–133. 10.1016/j.jbiomech.2011.09.022 22018682

[B7] Crenshaw J. R. Kaufman K. R. Grabiner M. D. (2013a). Compensatory-step training of healthy, mobile people with unilateral, transfemoral or knee disarticulation amputations: a potential intervention for trip-related falls. Gait and Posture 38, 500–506. 10.1016/j.gaitpost.2013.01.023 23433547

[B8] Crenshaw J. R. Kaufman K. R. Grabiner M. D. (2013b). Trip recoveries of people with unilateral, transfemoral or knee disarticulation amputations: initial findings. Gait and Posture 38, 534–536. 10.1016/j.gaitpost.2012.12.013 23369663

[B9] Culver S. C. Vailati L. G. Goldfarb M. (2022). “A primarily-passive knee prosthesis with powered stance and swing assistance,” in 2022 International Conference on Rehabilitation Robotics (ICORR) (IEEE), 1–6. 10.1109/ICORR55369.2022.9896545 36176112

[B10] Elery T. Rezazadeh S. Nesler C. Doan J. Zhu H. Gregg R. D. (2018). “Design and benchtop validation of a powered knee-ankle prosthesis with high-torque, low-impedance actuators,” in 2018 IEEE International Conference on Robotics and Automation (ICRA) (IEEE), 2788–2795. 10.1109/ICRA.2018.8461259 PMC6309177 30598854

[B11] Eng J. J. Winter D. A. Patla A. E. (1994). Strategies for recovery from a trip in early and late swing during human walking. Exp. Brain Research 102, 339–349. 10.1007/BF00227520 7705511

[B12] Eveld M. E. King S. T. Vailati L. G. Zelik K. E. Goldfarb M. (2021). On the basis for stumble recovery strategy selection in healthy adults. J. Biomechanical Engineering 143, 071003. 10.1115/1.4050171 33590838 PMC8086400

[B13] Fuenzalida Squella S. A. Kannenberg A. Brandão Benetti Â. (2018). Enhancement of a prosthetic knee with a microprocessor-controlled gait phase switch reduces falls and improves balance confidence and gait speed in community ambulators with unilateral transfemoral amputation. Prosthetics Orthotics International 42, 228–235. 10.1177/0309364617716207 28691574 PMC5888771

[B14] Gordon M. Thatte N. Geyer H. (2019). “Online learning for proactive obstacle avoidance with powered transfemoral prostheses,” in 2019 International Conference on Robotics and Automation (ICRA) (IEEE), 7920–7925.

[B15] Grabiner M. D. Bareither M. L. Gatts S. Marone J. Troy K. L. (2012). Task-specific training reduces trip-related fall risk in women 10.1249/MSS.0b013e318268c89f 22811033

[B16] Hafner B. J. Willingham L. L. Buell N. C. Allyn K. J. Smith D. G. (2007). Evaluation of function, performance, and preference as transfemoral amputees transition from mechanical to microprocessor control of the prosthetic knee. Archives Physical Medicine Rehabilitation 88, 207–217. 10.1016/j.apmr.2006.10.030 17270519

[B17] Highsmith M. J. Kahle J. T. Bongiorni D. R. Sutton B. S. Groer S. Kaufman K. R. (2010). Safety, energy efficiency, and cost efficacy of the c-leg for transfemoral amputees: a review of the literature. Prosthetics Orthotics International 34, 362–377. 10.3109/03093646.2010.520054 20969495

[B18] Kahle J. T. Highsmith M. J. Hubbard S. L. (2008). Comparison of nonmicroprocessor knee mechanism *versus* c-leg on prosthesis evaluation questionnaire, stumbles, falls, walking tests, stair descent, and knee preference. J. Rehabilitation Research Development 45, 1–14. 10.1682/jrrd.2007.04.0054 18566922

[B19] King S. T. Eveld M. E. Martínez A. Zelik K. E. Goldfarb M. (2019). A novel system for introducing precisely-controlled, unanticipated gait perturbations for the study of stumble recovery. J. Neuroengineering Rehabilitation 16, 1–17. 10.1186/s12984-019-0527-7 31182126 PMC6558741

[B20] King S. T. Eveld M. E. Zelik K. E. Goldfarb M. (2024). Factors leading to falls in transfemoral prosthesis users: a case series of prosthesis-side stumble recovery responses. J. Neuroengineering Rehabilitation 21, 117. 10.1186/s12984-024-01402-0 39003469 PMC11245817

[B21] King S. T. Eveld M. E. Vailati L. G. Zelik K. E. Goldfarb M. (2026). A preliminary study of the efficacy of bimodal *versus* unimodal stumble recovery responses in a powered knee prosthesis. Wearable Technol. 7, e4. 10.1017/wtc.2026.10040 41983188 PMC13071851

[B22] Lawson B. E. Varol H. A. Sup F. Goldfarb M. (2010). “Stumble detection and classification for an intelligent transfemoral prosthesis,” in *2010 Annual International Conference of the IEEE Engineering in Medicine and Biology* (IEEE), 511–514. 10.1109/IEMBS.2010.5626021 21095656

[B23] Lawson B. E. Mitchell J. Truex D. Shultz A. Ledoux E. Goldfarb M. (2014). A robotic leg prosthesis: design, control, and implementation. IEEE Robotics and Automation Mag. 21, 70–81. 10.1109/mra.2014.2360303

[B24] Lee J. T. Bartlett H. L. Goldfarb M. (2019). Design of a semipowered stance-control swing-assist transfemoral prosthesis. IEEE/ASME Trans. Mechatronics 25, 175–184. 10.1109/tmech.2019.2952084 33746502 PMC7977329

[B25] Lenzi T. Cempini M. Hargrove L. Kuiken T. (2018). Design, development, and testing of a lightweight hybrid robotic knee prosthesis. Int. J. Robotics Res. 37, 953–976. 10.1177/0278364918785993

[B26] Mendez J. Hood S. Gunnel A. Lenzi T. (2020). Powered knee and ankle prosthesis with indirect volitional swing control enables level-ground walking and crossing over obstacles. Sci. Robotics 5, eaba6635. 10.1126/scirobotics.aba6635 33022611 PMC8020725

[B27] O’Connor C. M. Thorpe S. K. O’Malley M. J. Vaughan C. L. (2007). Automatic detection of gait events using kinematic data. Gait and Posture 25, 469–474. 10.1016/j.gaitpost.2006.05.016 16876414

[B28] Pavol M. J. Owings T. M. Foley K. T. Grabiner M. D. (2001). Mechanisms leading to a fall from an induced trip in healthy older adults. Journals Gerontology Ser. A Biol. Sci. Med. Sci. 56, M428–M437. 10.1093/gerona/56.7.m428 11445602

[B29] Pijnappels M. Reeves N. D. Maganaris C. N. Van Dieen J. H. (2008). Tripping without falling; lower limb strength, a limitation for balance recovery and a target for training in the elderly. J. Electromyogr. Kinesiol. 18, 188–196. 10.1016/j.jelekin.2007.06.004 17761436

[B30] Rajasekaran V. Aranda J. Casals A. Pons J. L. (2015). An adaptive control strategy for postural stability using a wearable robot. Robotics Aut. Syst. 73, 16–23. 10.1016/j.robot.2014.11.014

[B31] Rezazadeh S. Quintero D. Divekar N. Reznick E. Gray L. Gregg R. D. (2019). A phase variable approach for improved rhythmic and non-rhythmic control of a powered knee-ankle prosthesis. IEEE Access 7, 109840–109855. 10.1109/ACCESS.2019.2933614 31656719 PMC6813797

[B32] Rosenblatt N. J. Marone J. Grabiner M. D. (2013). Preventing trip-related falls by community-dwelling adults: a prospective study. J. Am. Geriatrics Soc. 61, 1629–1631. 10.1111/jgs.12428 24028366

[B33] Rouse E. J. Mooney L. M. Martinez-Villalpando E. C. Herr H. M. (2013). “Clutchable series-elastic actuator: design of a robotic knee prosthesis for minimum energy consumption,” in 2013 IEEE 13th International Conference on Rehabilitation Robotics (ICORR) (IEEE), 1–6. 10.1109/ICORR.2013.6650383 24187202

[B34] Schillings A. Van Wezel B. Mulder T. Duysens J. (2000). Muscular responses and movement strategies during stumbling over obstacles. J. Neurophysiology 83, 2093–2102. 10.1152/jn.2000.83.4.2093 10758119

[B35] Shirota C. Simon A. M. Kuiken T. A. (2015). Transfemoral amputee recovery strategies following trips to their sound and prosthesis sides throughout swing phase. J. Neuroengineering Rehabilitation 12, 1–11. 10.1186/s12984-015-0067-8 26353775 PMC4564965

[B36] Sup F. Varol H. A. Mitchell J. Withrow T. J. Goldfarb M. (2009). Preliminary evaluations of a self-contained anthropomorphic transfemoral prosthesis. IEEE/ASME Trans. Mechatronics 14, 667–676. 10.1109/TMECH.2009.2032688 20054424 PMC2801882

[B37] Talbot L. A. Musiol R. J. Witham E. K. Metter E. J. (2005). Falls in young, middle-aged and older community dwelling adults: perceived cause, environmental factors and injury. BMC Public Health 5, 1–9. 10.1186/1471-2458-5-86 16109159 PMC1208908

[B38] Thatte N. Geyer H. (2015). Toward balance recovery with leg prostheses using neuromuscular model control. IEEE Trans. Biomed. Eng. 63, 904–913. 10.1109/TBME.2015.2472533 26315935 PMC4854805

[B39] Thatte N. Srinivasan N. Geyer H. (2019). Real-time reactive trip avoidance for powered transfemoral prostheses. Robotics Sci. Syst. 10.15607/RSS.2019.XV.034

[B40] Tran M. Gabert L. Cempini M. Lenzi T. (2019). A lightweight, efficient fully powered knee prosthesis with actively variable transmission. IEEE Robotics Automation Lett. 4, 1186–1193. 10.1109/lra.2019.2892204

[B41] Verma S. K. Willetts J. L. Corns H. L. Marucci-Wellman H. R. Lombardi D. A. Courtney T. K. (2016). Falls and fall-related injuries among community-dwelling adults in the United States. PLoS One 11, e0150939. 10.1371/journal.pone.0150939 26977599 PMC4792421

[B42] Zhang F. D’andrea S. E. Nunnery M. J. Kay S. M. Huang H. (2011). Towards design of a stumble detection system for artificial legs. IEEE Transactions Neural Systems Rehabilitation Engineering 19, 567–577. 10.1109/TNSRE.2011.2161888 PMC3192236 21859635

[B43] Zhu W. Cai M. Hou H. Zhao S. Ruan L. Wang Q. (2025). “Obstacle avoidance for knee prostheses *via* direct integration of environment information,” in 2025 44th Chinese Control Conference (CCC) (IEEE), 5058–5063.

